# 5-Bromo-3-cyclo­pentyl­sulfinyl-2-methyl-1-benzofuran

**DOI:** 10.1107/S1600536811017120

**Published:** 2011-05-11

**Authors:** Pil Ja Seo, Hong Dae Choi, Byeng Wha Son, Uk Lee

**Affiliations:** a Department of Chemistry, Dongeui University, San 24 Kaya-dong Busanjin-gu, Busan 614-714, Republic of Korea; b Department of Chemistry, Pukyong National University, 599-1 Daeyeon 3-dong, Nam-gu, Busan 608-737, Republic of Korea

## Abstract

In the title compound, C_14_H_15_BrO_2_S, the cyclo­pentyl ring adopts an envelope conformation. In the cyclo­pentyl ring, two adjacent C atoms are disordered over two sets of sites with site-occupancy factors of 0.618 (11) and 0.382 (11). In the crystal, mol­ecules are linked through weak inter­molecular C—H⋯O hydrogen bonds.

## Related literature

For the biological activity of benzofuran compounds, see: Aslam *et al.* (2009 ▶); Galal *et al.* (2009 ▶); Khan *et al.* (2005 ▶). For natural products with benzofuran rings, see: Akgul & Anil (2003 ▶); Soekamto *et al.* (2003 ▶). For structural studies of related 5-bromo-3-cyclo­hexyl­sulfinyl-2-methyl-1-benzofuran derivatives, see: Choi *et al.* (2011**a* ▶,b* ▶).
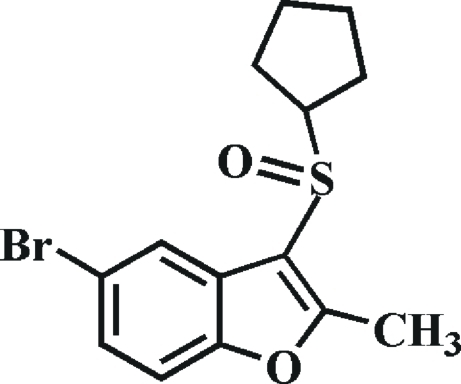


## Experimental

### 

#### Crystal data

C_14_H_15_BrO_2_S
*M* *_r_* = 327.23
Monoclinic, 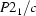

*a* = 9.4166 (6) Å
*b* = 9.5369 (7) Å
*c* = 15.1662 (11) Å
β = 100.033 (4)°
*V* = 1341.17 (16) Å^3^
*Z* = 4
Mo *K*α radiation
μ = 3.21 mm^−1^
*T* = 173 K
0.31 × 0.24 × 0.13 mm

#### Data collection

Bruker SMART APEXII CCD diffractometer
Absorption correction: multi-scan (*SADABS*; Bruker, 2009 ▶) *T*_min_ = 0.531, *T*_max_ = 0.746
12376 measured reflections
3098 independent reflections
2567 reflections with *I* > 2σ(*I*)
*R*_int_ = 0.035

#### Refinement

*R*[*F* ^2^ > 2σ(*F* ^2^)] = 0.035
*wR*(*F* ^2^) = 0.094
*S* = 1.06
3098 reflections
182 parameters
83 restraints
H-atom parameters constrained
Δρ_max_ = 0.54 e Å^−3^
Δρ_min_ = −0.64 e Å^−3^

### 

Data collection: *APEX2* (Bruker, 2009 ▶); cell refinement: *SAINT* (Bruker, 2009 ▶); data reduction: *SAINT*; program(s) used to solve structure: *SHELXS97* (Sheldrick, 2008 ▶); program(s) used to refine structure: *SHELXL97* (Sheldrick, 2008 ▶); molecular graphics: *ORTEP-3* (Farrugia, 1997 ▶) and *DIAMOND* (Brandenburg, 1998 ▶); software used to prepare material for publication: *SHELXL97*.

## Supplementary Material

Crystal structure: contains datablocks global, I. DOI: 10.1107/S1600536811017120/qk2009sup1.cif

Structure factors: contains datablocks I. DOI: 10.1107/S1600536811017120/qk2009Isup2.hkl

Supplementary material file. DOI: 10.1107/S1600536811017120/qk2009Isup3.cml

Additional supplementary materials:  crystallographic information; 3D view; checkCIF report

## Figures and Tables

**Table 1:** Hydrogen-bond geometry (Å, °)

*D*—H⋯*A*	*D*—H	H⋯*A*	*D*⋯*A*	*D*—H⋯*A*
C3—H3⋯O2^i^	0.95	2.46	3.392 (3)	169

Symmetry code: (i) 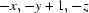.

**Fig. 1.**
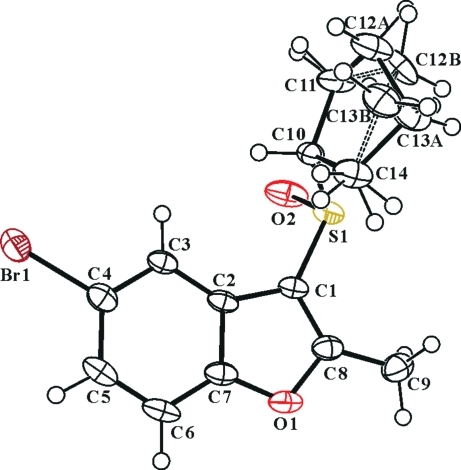
The molecular structure of the title compound with the atom numbering scheme. Displacement ellipsoids are drawn at the 50% probability level. H atoms are presented as a small spheres of arbitrary radius.

## supplementary crystallographic information

### Comment

Recently, many compounds having a benzofuran skeleton have attracted much attention due to their diverse pharmacological properties such as antibacterial and antifungal, antitumor and antiviral, and antimicrobial activities (Aslam *et al.*, 2009, Galal *et al.*, 2009, Khan *et al.*, 2005). These benzofuran derivatives occur in a wide range of natural products (Akgul & Anil, 2003; Soekamto *et al.*, 2003). As part of our ongoing program of the substituent effect on the solid state structures of 5-bromo-3-cyclohexylsulfinyl-2-methyl-1-benzofuran analogues (Choi *et al.*, 2011**a*, b*), we report herein the crystal structure of the title compound.

In the title molecule (Fig. 1), the benzofuran unit is essentially planar, with a mean deviation of 0.012 (2) Å from the least-squares plane defined by the nine constituent atoms. The cyclopentyl ring is in the envelope form. In the cyclopentyl ring, two C atoms (C12 & C13) are disordered over two positions with site-occupancy factors, from refinement of 0.62 (1) (part A) and 0.38 (1) (part B). The crystal packing is stabilized by weak intermolecular C—H···O hydrogen bonds; the first one between a benzene H atom and the O atom of the sulfinyl group (Table 1; C3—H3···O2^i^), and the second one between a cyclopentyl H atom and the furan O atom (Table 1; C11—H11C···O1^ii^).

### Experimental

77% 3-chloroperoxybenzoic acid (269 mg, 1.2 mmol) was added in small portions to a stirred solution of 5-bromo-3-cyclopentylsulfanyl-2-methyl-1-benzofuran (373 mg, 1.2 mmol) in dichloromethane (30 mL) at 273 K. After being stirred at room temperature for 4h, the mixture was washed with saturated sodium bicarbonate solution and the organic layer was separated, dried over anhydrous magnesium sulfate, filtered and concentrated at reduced pressure. The residue was purified by column chromatography (hexane–ethyl acetate, 1:1 v/v) to afford the title compound as a colorless solid [yield 71%, m.p. 405–406 K; *R*_f_ = 0.66 (hexane–ethyl acetate, 1:1 v/v)]. Single crystals suitable for X-ray diffraction were prepared by slow evaporation of a solution of the title compound in ethyl acetate at room temperature.

### Refinement

All H atoms were positioned geometrically and refined using a riding model, with C—H = 0.95 Å for aryl, 1.00 Å for methine, 0.99 Å for methylene and 0.98 Å for methyl H atoms, respectively. *U*_iso_(H) = 1.2*U*_eq_(C) for aryl, methine, and methylene, and 1.5*U*_eq_(C) for methyl H atoms. Two C atoms of the cyclopentyl ring are disordered over two positions with site-occupancy factors, from refinement of 0.62 (1) (part A) and 0.38 (1) (part B). The distance of equivalent C–C pairs was restrained to 0.001 Å using commd  SADI and DELU, and displacement ellipsoids of C12 and C13 set were restrained to 0.01 using command ISOR.

### Crystal data

**Table:**

C_14_H_15_BrO_2_S	*F*(000) = 664
*M**_r_* = 327.23	*D*_x_ = 1.621 Mg m^−^^3^
Monoclinic, *P*2_1_/*c*	Mo *K*α radiation, λ = 0.71073 Å
Hall symbol: -P 2ybc	Cell parameters from 5053 reflections
*a* = 9.4166 (6) Å	θ = 2.5–27.4°
*b* = 9.5369 (7) Å	µ = 3.21 mm^−^^1^
*c* = 15.1662 (11) Å	*T* = 173 K
β = 100.033 (4)°	Block, colourless
*V* = 1341.17 (16) Å^3^	0.31 × 0.24 × 0.13 mm
*Z* = 4	

### Data collection

**Table:**

Bruker SMART APEXII CCD diffractometer	3098 independent reflections
Radiation source: rotating anode	2567 reflections with *I* > 2σ(*I*)
graphite multilayer	*R*_int_ = 0.035
Detector resolution: 10.0 pixels mm^-1^	θ_max_ = 27.6°, θ_min_ = 2.2°
φ and ω scans	*h* = −12→12
Absorption correction: multi-scan (*SADABS*; Bruker, 2009)	*k* = −11→12
*T*_min_ = 0.531, *T*_max_ = 0.746	*l* = −19→18
12376 measured reflections	

### Refinement

**Table:**

Refinement on *F*^2^	Primary atom site location: structure-invariant direct methods
Least-squares matrix: full	Secondary atom site location: difference Fourier map
*R*[*F*^2^ > 2σ(*F*^2^)] = 0.035	Hydrogen site location: difference Fourier map
*wR*(*F*^2^) = 0.094	H-atom parameters constrained
*S* = 1.06	*w* = 1/[σ^2^(*F*_o_^2^) + (0.0474*P*)^2^ + 0.9434*P*] where *P* = (*F*_o_^2^ + 2*F*_c_^2^)/3
3098 reflections	(Δ/σ)_max_ < 0.001
182 parameters	Δρ_max_ = 0.54 e Å^−^^3^
83 restraints	Δρ_min_ = −0.64 e Å^−^^3^

### Special details

**Table:**

Geometry. All esds (except the esd in the dihedral angle between two l.s. planes) are estimated using the full covariance matrix. The cell esds are taken into account individually in the estimation of esds in distances, angles and torsion angles; correlations between esds in cell parameters are only used when they are defined by crystal symmetry. An approximate (isotropic) treatment of cell esds is used for estimating esds involving l.s. planes.
Refinement. Refinement of F^2^ against ALL reflections. The weighted R-factor wR and goodness of fit S are based on F^2^, conventional R-factors R are based on F, with F set to zero for negative F^2^. The threshold expression of F^2^ > 2sigma(F^2^) is used only for calculating R-factors(gt) etc. and is not relevant to the choice of reflections for refinement. R-factors based on F^2^ are statistically about twice as large as those based on F, and R- factors based on ALL data will be even larger.

### Fractional atomic coordinates and isotropic or equivalent isotropic displacement parameters (Å^2^)

**Table:**

	*x*	*y*	*z*	*U*_iso_*/*U*_eq_	Occ. (<1)
Br1	0.23192 (3)	0.05192 (3)	0.013362 (19)	0.03823 (12)	
S1	0.16243 (6)	0.69180 (7)	0.10017 (4)	0.02871 (16)	
O1	0.56646 (18)	0.5680 (2)	0.13602 (12)	0.0323 (4)	
O2	0.0936 (2)	0.6788 (3)	0.00457 (13)	0.0467 (6)	
C1	0.3266 (2)	0.5992 (3)	0.11348 (16)	0.0255 (5)	
C2	0.3530 (2)	0.4544 (3)	0.09134 (15)	0.0243 (5)	
C3	0.2690 (3)	0.3376 (3)	0.06233 (15)	0.0250 (5)	
H3	0.1667	0.3424	0.0503	0.030*	
C4	0.3415 (3)	0.2147 (3)	0.05198 (16)	0.0290 (5)	
C5	0.4922 (3)	0.2047 (3)	0.06733 (18)	0.0340 (6)	
H5	0.5372	0.1176	0.0593	0.041*	
C6	0.5749 (3)	0.3209 (3)	0.09404 (18)	0.0348 (6)	
H6	0.6772	0.3172	0.1033	0.042*	
C7	0.5034 (3)	0.4422 (3)	0.10677 (17)	0.0283 (5)	
C8	0.4561 (3)	0.6612 (3)	0.13951 (16)	0.0292 (5)	
C9	0.5022 (3)	0.8028 (3)	0.17033 (19)	0.0376 (6)	
H9A	0.6078	0.8069	0.1833	0.056*	
H9B	0.4637	0.8250	0.2247	0.056*	
H9C	0.4659	0.8711	0.1235	0.056*	
C10	0.0670 (3)	0.5783 (3)	0.16509 (16)	0.0253 (5)	
H10	0.0627	0.4807	0.1406	0.030*	
C11	−0.0865 (3)	0.6359 (4)	0.16324 (19)	0.0395 (7)	
H11A	−0.0923	0.7357	0.1451	0.047*	0.618 (11)
H11B	−0.1578	0.5818	0.1209	0.047*	0.618 (11)
H11C	−0.1601	0.5618	0.1472	0.047*	0.382 (11)
H11D	−0.1074	0.7143	0.1202	0.047*	0.382 (11)
C12A	−0.1140 (5)	0.6192 (9)	0.2584 (3)	0.0431 (17)	0.618 (11)
H12A	−0.1388	0.5213	0.2713	0.052*	0.618 (11)
H12B	−0.1909	0.6829	0.2711	0.052*	0.618 (11)
C13A	0.0344 (5)	0.6615 (8)	0.3103 (4)	0.0475 (18)	0.618 (11)
H13A	0.0502	0.7637	0.3060	0.057*	0.618 (11)
H13B	0.0446	0.6349	0.3742	0.057*	0.618 (11)
C12B	−0.0817 (14)	0.6861 (9)	0.2588 (3)	0.044 (3)	0.382 (11)
H12C	−0.1795	0.6854	0.2744	0.053*	0.382 (11)
H12D	−0.0426	0.7826	0.2663	0.053*	0.382 (11)
C13B	0.0173 (8)	0.5832 (11)	0.3181 (6)	0.040 (2)	0.382 (11)
H13C	0.0495	0.6203	0.3793	0.049*	0.382 (11)
H13D	−0.0283	0.4903	0.3213	0.049*	0.382 (11)
C14	0.1399 (3)	0.5790 (3)	0.26381 (18)	0.0381 (7)	
H14A	0.1530	0.4823	0.2877	0.046*	0.618 (11)
H14B	0.2351	0.6259	0.2715	0.046*	0.618 (11)
H14C	0.1994	0.4937	0.2784	0.046*	0.382 (11)
H14D	0.2026	0.6624	0.2768	0.046*	0.382 (11)

### Atomic displacement parameters (Å^2^)

**Table:**

	*U*^11^	*U*^22^	*U*^33^	*U*^12^	*U*^13^	*U*^23^
Br1	0.04047 (18)	0.03448 (17)	0.04127 (18)	0.00614 (12)	0.01135 (13)	−0.00446 (12)
S1	0.0221 (3)	0.0325 (3)	0.0328 (3)	0.0048 (2)	0.0086 (2)	0.0075 (3)
O1	0.0183 (8)	0.0479 (12)	0.0312 (10)	0.0003 (8)	0.0054 (7)	0.0023 (8)
O2	0.0290 (10)	0.0792 (16)	0.0320 (10)	0.0134 (10)	0.0054 (8)	0.0183 (11)
C1	0.0188 (11)	0.0333 (13)	0.0256 (12)	0.0028 (10)	0.0073 (9)	0.0053 (10)
C2	0.0192 (11)	0.0359 (13)	0.0187 (11)	0.0060 (9)	0.0056 (9)	0.0062 (9)
C3	0.0208 (11)	0.0350 (13)	0.0202 (11)	0.0050 (10)	0.0062 (9)	0.0037 (10)
C4	0.0308 (13)	0.0357 (14)	0.0218 (12)	0.0059 (11)	0.0084 (10)	0.0018 (10)
C5	0.0314 (13)	0.0419 (15)	0.0310 (13)	0.0167 (12)	0.0118 (11)	0.0057 (12)
C6	0.0201 (11)	0.0531 (17)	0.0328 (14)	0.0121 (11)	0.0090 (10)	0.0069 (13)
C7	0.0207 (11)	0.0419 (15)	0.0232 (12)	0.0026 (10)	0.0064 (9)	0.0055 (11)
C8	0.0242 (12)	0.0405 (14)	0.0238 (12)	−0.0006 (11)	0.0060 (10)	0.0036 (11)
C9	0.0325 (14)	0.0440 (16)	0.0367 (15)	−0.0079 (12)	0.0068 (12)	−0.0013 (13)
C10	0.0208 (11)	0.0301 (13)	0.0266 (12)	−0.0003 (9)	0.0081 (9)	−0.0013 (10)
C11	0.0199 (12)	0.068 (2)	0.0320 (14)	0.0022 (13)	0.0071 (11)	0.0020 (14)
C12A	0.028 (2)	0.071 (5)	0.033 (3)	0.011 (3)	0.0120 (19)	0.005 (3)
C13A	0.037 (3)	0.081 (5)	0.025 (2)	0.008 (3)	0.006 (2)	−0.009 (3)
C12B	0.054 (6)	0.049 (5)	0.035 (4)	0.022 (4)	0.027 (4)	0.006 (3)
C13B	0.044 (4)	0.048 (5)	0.034 (4)	0.004 (4)	0.019 (3)	0.007 (4)
C14	0.0281 (13)	0.0590 (19)	0.0271 (13)	0.0070 (13)	0.0049 (11)	0.0073 (13)

### Geometric parameters (Å, °)

**Table:**

Br1—C4	1.900 (3)	C10—H10	1.0000
S1—O2	1.487 (2)	C11—C12A	1.519 (4)
S1—C1	1.761 (2)	C11—C12B	1.519 (4)
S1—C10	1.805 (2)	C11—H11A	0.9900
O1—C8	1.375 (3)	C11—H11B	0.9900
O1—C7	1.377 (3)	C11—H11C	0.9900
C1—C8	1.351 (4)	C11—H11D	0.9900
C1—C2	1.453 (4)	C12A—C13A	1.533 (6)
C2—C3	1.393 (4)	C12A—H12A	0.9900
C2—C7	1.399 (3)	C12A—H12B	0.9900
C3—C4	1.379 (3)	C13A—C14	1.532 (4)
C3—H3	0.9500	C13A—H13A	0.9900
C4—C5	1.400 (4)	C13A—H13B	0.9900
C5—C6	1.374 (4)	C12B—C13B	1.533 (6)
C5—H5	0.9500	C12B—H12C	0.9900
C6—C7	1.369 (4)	C12B—H12D	0.9900
C6—H6	0.9500	C13B—C14	1.531 (4)
C8—C9	1.471 (4)	C13B—H13C	0.9900
C9—H9A	0.9800	C13B—H13D	0.9900
C9—H9B	0.9800	C14—H14A	0.9900
C9—H9C	0.9800	C14—H14B	0.9900
C10—C14	1.535 (4)	C14—H14C	0.9900
C10—C11	1.541 (3)	C14—H14D	0.9900
			
O2—S1—C1	107.12 (12)	C10—C11—H11C	111.4
O2—S1—C10	107.95 (12)	H11A—C11—H11C	128.0
C1—S1—C10	98.47 (11)	C12A—C11—H11D	131.3
C8—O1—C7	106.76 (19)	C12B—C11—H11D	111.1
C8—C1—C2	107.5 (2)	C10—C11—H11D	111.0
C8—C1—S1	122.9 (2)	H11A—C11—H11D	25.2
C2—C1—S1	129.26 (19)	H11B—C11—H11D	86.0
C3—C2—C7	119.3 (2)	H11C—C11—H11D	109.1
C3—C2—C1	136.2 (2)	C11—C12A—C13A	99.7 (4)
C7—C2—C1	104.5 (2)	C11—C12A—H12A	111.8
C4—C3—C2	116.7 (2)	C13A—C12A—H12A	111.8
C4—C3—H3	121.6	C11—C12A—H12B	111.8
C2—C3—H3	121.6	C13A—C12A—H12B	111.8
C3—C4—C5	123.1 (3)	H12A—C12A—H12B	109.6
C3—C4—Br1	118.46 (19)	C14—C13A—C12A	103.6 (4)
C5—C4—Br1	118.4 (2)	C14—C13A—H13A	111.0
C6—C5—C4	120.0 (2)	C12A—C13A—H13A	111.0
C6—C5—H5	120.0	C14—C13A—H13B	111.0
C4—C5—H5	120.0	C12A—C13A—H13B	111.0
C7—C6—C5	117.1 (2)	H13A—C13A—H13B	109.0
C7—C6—H6	121.5	C11—C12B—C13B	105.8 (5)
C5—C6—H6	121.5	C11—C12B—H12C	110.6
C6—C7—O1	125.9 (2)	C13B—C12B—H12C	110.6
C6—C7—C2	123.7 (3)	C11—C12B—H12D	110.6
O1—C7—C2	110.4 (2)	C13B—C12B—H12D	110.6
C1—C8—O1	110.9 (2)	H12C—C12B—H12D	108.7
C1—C8—C9	134.2 (2)	C14—C13B—C12B	98.0 (5)
O1—C8—C9	115.0 (2)	C14—C13B—H13C	112.2
C8—C9—H9A	109.5	C12B—C13B—H13C	112.2
C8—C9—H9B	109.5	C14—C13B—H13D	112.2
H9A—C9—H9B	109.5	C12B—C13B—H13D	112.2
C8—C9—H9C	109.5	H13C—C13B—H13D	109.8
H9A—C9—H9C	109.5	C13B—C14—C10	105.9 (4)
H9B—C9—H9C	109.5	C13A—C14—C10	103.6 (3)
C14—C10—C11	106.0 (2)	C13B—C14—H14A	83.4
C14—C10—S1	110.42 (18)	C13A—C14—H14A	111.0
C11—C10—S1	109.34 (19)	C10—C14—H14A	111.0
C14—C10—H10	110.3	C13B—C14—H14B	132.5
C11—C10—H10	110.3	C13A—C14—H14B	111.0
S1—C10—H10	110.3	C10—C14—H14B	111.0
C12A—C11—C10	105.1 (3)	H14A—C14—H14B	109.0
C12B—C11—C10	102.9 (5)	C13B—C14—H14C	110.6
C12A—C11—H11A	110.7	C13A—C14—H14C	134.9
C12B—C11—H11A	87.3	C10—C14—H14C	110.8
C10—C11—H11A	110.7	H14B—C14—H14C	83.1
C12A—C11—H11B	110.7	C13B—C14—H14D	110.5
C12B—C11—H11B	133.5	C13A—C14—H14D	84.7
C10—C11—H11B	110.7	C10—C14—H14D	110.4
H11A—C11—H11B	108.8	H14A—C14—H14D	130.1
C12A—C11—H11C	86.0	H14C—C14—H14D	108.7
C12B—C11—H11C	111.2		
			
O2—S1—C1—C8	118.2 (2)	S1—C1—C8—C9	6.3 (4)
C10—S1—C1—C8	−129.9 (2)	C7—O1—C8—C1	−0.2 (3)
O2—S1—C1—C2	−54.7 (2)	C7—O1—C8—C9	179.5 (2)
C10—S1—C1—C2	57.2 (2)	O2—S1—C10—C14	175.88 (18)
C8—C1—C2—C3	178.9 (3)	C1—S1—C10—C14	64.7 (2)
S1—C1—C2—C3	−7.3 (4)	O2—S1—C10—C11	−67.9 (2)
C8—C1—C2—C7	−0.1 (3)	C1—S1—C10—C11	−179.06 (19)
S1—C1—C2—C7	173.68 (19)	C14—C10—C11—C12A	−19.1 (4)
C7—C2—C3—C4	1.0 (3)	S1—C10—C11—C12A	−138.2 (4)
C1—C2—C3—C4	−177.9 (3)	C14—C10—C11—C12B	8.5 (5)
C2—C3—C4—C5	−1.4 (3)	S1—C10—C11—C12B	−110.5 (5)
C2—C3—C4—Br1	179.56 (17)	C12B—C11—C12A—C13A	−48.7 (8)
C3—C4—C5—C6	0.0 (4)	C10—C11—C12A—C13A	40.2 (7)
Br1—C4—C5—C6	179.0 (2)	C11—C12A—C13A—C14	−46.9 (8)
C4—C5—C6—C7	1.7 (4)	C12A—C11—C12B—C13B	63.3 (8)
C5—C6—C7—O1	177.8 (2)	C10—C11—C12B—C13B	−34.7 (10)
C5—C6—C7—C2	−2.2 (4)	C11—C12B—C13B—C14	46.2 (11)
C8—O1—C7—C6	−179.9 (2)	C12B—C13B—C14—C13A	49.8 (6)
C8—O1—C7—C2	0.1 (3)	C12B—C13B—C14—C10	−39.8 (9)
C3—C2—C7—C6	0.8 (4)	C12A—C13A—C14—C13B	−62.9 (8)
C1—C2—C7—C6	180.0 (2)	C12A—C13A—C14—C10	35.4 (7)
C3—C2—C7—O1	−179.2 (2)	C11—C10—C14—C13B	20.3 (5)
C1—C2—C7—O1	0.0 (3)	S1—C10—C14—C13B	138.6 (5)
C2—C1—C8—O1	0.1 (3)	C11—C10—C14—C13A	−10.0 (4)
S1—C1—C8—O1	−174.09 (16)	S1—C10—C14—C13A	108.3 (4)
C2—C1—C8—C9	−179.5 (3)		

### Hydrogen-bond geometry (Å, °)

**Table:**

*D*—H···*A*	*D*—H	H···*A*	*D*···*A*	*D*—H···*A*
C3—H3···O2^i^	0.95	2.46	3.392 (3)	169

Symmetry codes: (i) −*x*, −*y*+1, −*z*.
